# Interactive association of type 2 diabetes and subclinical CeVD markers with cognitive decline and incident dementia: A memory‐clinic study

**DOI:** 10.1002/alz.087538

**Published:** 2025-01-03

**Authors:** Jiangbo Cui, Christopher Chen, Saima Hilal

**Affiliations:** ^1^ Yong Loo Lin School of Medicine, National University of Singapore, Singapore Singapore; ^2^ Saw Swee Hock School of Public Health, National University of Singapore and National University Health System, Singapore, Singapore Singapore

## Abstract

**Background:**

Association of type 2 diabetes with cognitive decline and incident dementia in older adults remains inconsistent. In this study, we aim to investigate whether subclinical cerebrovascular disease (sCeVD) would modify this relationship.

**Method:**

A total of 654 participants underwent brain MRI at baseline, from whom 592 individuals with at least one follow‐up were selected for longitudinal analysis. Cognitive assessments, including Clinical Dementia Rating Sum‐of‐boxes (CDR‐SOB) and detailed neuropsychological tests, were performed annually for up to 5 years. sCeVD markers of interest were lacunes, white matter hyperintensities (WMHs), cerebral microbleeds (CMBs), intracranial stenosis (ICS), and cortical infarcts.

**Result:**

Diabetes was associated with presence of lacunes, WMHs and ICS in multivariate‐adjusted model. Notably, a significant interaction term (diabetes × WMHs × time) was observed, especially among stroke‐free individuals, suggesting an interactive effect on cognition. Subgroup analyses showed association of diabetes with cognitive decline was stronger in participants with high WMHs but not significant in those with low WMHs. Moreover, participants with both diabetes and high WMHs showed the greatest cognitive decline in CDR‐SOB, global cognition, language, and memory, along with the highest risk of dementia.

**Conclusion:**

Older adults with diabetes compared those without had more sCeVD lesions, and the impact of diabetes on cognitive decline is contingent on the presence of high load WMHs. Our findings suggested that WMHs could be used for risk stratification among older adults with diabetes.

